# Detection of Adulteration in Infant Formula Based on Ensemble Convolutional Neural Network and Near-Infrared Spectroscopy

**DOI:** 10.3390/foods10040785

**Published:** 2021-04-06

**Authors:** Yisen Liu, Songbin Zhou, Wei Han, Chang Li, Weixin Liu, Zefan Qiu, Hong Chen

**Affiliations:** Guangdong Key Laboratory of Modern Control Technology, Guangdong Academy of Sciences, Institute of Intelligent Manufacturing, Guangzhou 510070, China; ys.liu@giim.ac.cn (Y.L.); w.han@giim.ac.cn (W.H.); c.li@giim.ac.cn (C.L.); wx.liu@giim.ac.cn (W.L.); zf.qiu@giim.ac.cn (Z.Q.); chen.h@giim.ac.cn (H.C.)

**Keywords:** infant formula adulteration, ensemble learning, convolutional neural network, wavelength selection, attention mechanism

## Abstract

Adulteration in dairy products has received world-wide attention, and at the same time, near infrared (NIR) spectroscopy has proven to be a promising tool for adulteration detection given its advantages of real-time response and non-destructive analysis. Regardless, the accurate and robust NIR model for adulteration detection is hard to achieve in practice. Convolutional neural network (CNN), as a promising deep learning architecture, is difficult to apply to such chemometrics tasks due to the high risk of overfitting, despite the breakthroughs it has made in other fields. In this paper, the ensemble learning method based on CNN estimators was developed to address the overfitting and random initialization problems of CNN and applied to the determination of two infant formula adulterants, namely hydrolyzed leather protein (HLP) and melamine. Moreover, a probabilistic wavelength selection method based on the attention mechanism was proposed for the purpose of finding the best trade-off between the accuracy and the diversity of the sub-models in ensemble learning. The overall results demonstrate that the proposed method yielded superiority regression performance over the comparison methods for both studied data sets, and determination coefficients (R^2^) of 0.961 and 0.995 were obtained for the HLP and the melamine data sets, respectively.

## 1. Introduction

Adulteration in dairy products has received wide attention from the international community [1,2,3], especially after the global food safety scares caused by melamine adulteration in 2008, because it not only compromises the nutritional quality of the dairy products, but also brings significant health risks to the consumers. There are two typical types of adulterants for dairy products, one of which is mainly used to reduce the production cost, such as starch and maltodextrin, and the other is attempted to boost the apparent protein content, including melamine, hydrolyzed leather protein (HLP), urea, etc. 

An effective detection method is crucial for the control of dairy product adulteration. For instance, the determination of melamine and hydrolyzed leather protein cannot be achieved by the widely-used Kjeldahl method [4], because it is a nonspecific procedure that determines the protein content by quantifying the presence of nitrogen. Improved methods [5] were proposed for the separation of non-protein nitrogen from true protein nitrogen by adding some protein precipitating agents during the Kjeldahl test. However, the detection accuracy still needs to be improved since the composition and the amount of non-protein nitrogen are susceptible to the type and concentration of protein precipitation [6]. Based on this circumstance, a number of high-precision detection techniques have been developed. One of the most concerned approaches for adulterants detection is the chromatography-based methods, such as high-performance liquid chromatography (HPLC) [7], gas chromatography-tandem mass spectrometry (GC-MS) [8], capillary electrophoresis-mass spectrometry (CE-MS) [9] and micellar electrokinetic chromatography (MEKC) [10]. Other analytical chemistry methods, such as enzyme-linked immunosorbent assay (ELISA) [11], polymerase chain reaction (PCR) [12], optical biosensor [13] and spectroscopic fluorescence-based techniques [14] were also developed to detect different kinds of adulterants in dairy products. Most of these methods are highly sensitive (detection limits reach ppm level), but usually time-consuming, expensive, and require complicated sample pretreatments and well-trained technicians. Compared with the above methods, near-infrared (NIR) spectroscopy is an analytical technique that has advantages in real-time response, simplicity in testing, relatively low-cost instrument, non-destructive behavior and environmentally friendly analysis [15,16,17]. 

Quite a few efforts have been made on chemometric algorithms to achieve better detection accuracy of dairy product adulteration based on NIR spectroscopy [18,19,20]. For instance, Borin et al. adopted least-squares support vector machine (LS-SVM) for the simultaneous quantification of some common adulterants (starch, whey or sucrose) found in milk powder [21]. Balabin and Smirnov found that the nonlinear calibration methods provided better results when concerning predicting melamine in dairy products, after comparing the performance of partial least squares regression (PLS), polynomial PLS, artificial neural network (ANN), support vector regression (SVR) and least squares support vector machine (LS-SVM) [22]. Mabood et al. developed PLS-DA and PLS models for the qualitative and quantitative analysis of adulteration in camel milk with goat milk [23]. 

Convolutional neural network (CNN) [24], which is one of the important network architectures developed in the ongoing deep learning revolution, has begun to show impact on chemometrics in recent years [25,26,27,28]. Acquarelli et al. employed the CNN for classification of vibrational spectroscopy (Raman, NIR and FT-IR) and developed a retraining algorithm for the selection of important wavelengths [29]. Cui et al. used the CNN for NIR data regression and found that the convolutional filter can automatically accomplish the suitable spectral preprocessing [30]. Chen et al. divided the NIR spectra into several subintervals as the inputs of CNN models and applied genetic algorithm (GA) to optimize the parameters of each subinterval model, which generates more representative features than fixed convolutional parameters [31]. In addition to the qualitative and quantitative approaches mentioned above, CNN was also employed for super-resolution of spectra image [32,33], peak detection [34], and denoising [35] of spectrometry data. All these recent studies demonstrate that the spectroscopy has greatly benefited from the involvement of CNN given its advantages in strong comprehensive ability, requiring less prior knowledge for the data set, easy to integrate the spectral and the morphology information, and capable of finding effective bands [36].

Despite the promising results of the CNN in processing NIR spectral data, challenges remain due to the high risk of overfitting. For the chemometrics tasks, it is hard to obtain labeled sample sets in a large size because of higher sample preparing and testing costs when compared with the computer vision tasks. Various strategies, such as data augmentation [37] and shallow CNN [29], have been proposed by the researchers to avoid the overfitting issue in spectral modeling. Another concern is that the stability and robustness of the CNN-based models need to be improved because of the random initial value of the CNN weights. The random initial weights lead to different optimal trajectories in each training process, thus resulting in unstable predicted values.

Ensemble learning, which combines a number of base estimators to achieve better predictive performance [38,39,40], is an appropriate candidate for addressing the overfitting and random initialization problems of CNN. In the former studies, most of the ensemble learning approaches for NIR spectra processing adopted PLS, which is a classical linear regressor, as the base estimators [41,42,43]. Popular ensemble strategies, including bagging [44], boosting [45] and stacking [46], were employed to integrate and improve the predicting results of the basic PLS models. Zhou et al. compared bagging-PLS with boosting-PLS in online near-infrared models for monitoring active pharmaceutical ingredients of Chinese Medicine [47]. Bi et al. proposed a dual-stacked-PLS algorithm and compared several combination rules of the outer stack step [48]. There are also some ensemble learning methods based on other nonlinear classifiers or regressors, such as the extreme learning machine (ELM) [49] and the support vector machine (SVM) [50]. To the best of our knowledge, the ensemble learning method based on CNN for spectrometric analysis is rarely reported and needs to be further studied.

In this paper, we proposed an ensemble-CNN algorithm for the quantitative analysis of adulteration in infant formula based on NIR spectroscopy. Besides, we designed a wavelength selection strategy based on the attention mechanism for the proposed ensemble architecture. It should be mentioned that a good ensemble learner requires sub-models with good accuracy and diversity. Different wavelength selection strategies have been adopted by the former ensemble architectures in order to improve the accuracy or the diversity of the sub-models. Random selection of variable subspaces [39,51] is a frequently-used strategy to promote the diversity, however, may result in some poorly performing sub-models, given the selection of some inefficient wavelength combinations. On the other hand, several wavelength selection methods, including SPA [52], uninformative variable elimination (UVE) [41] and synergy interval partial least squares algorithm (SiPLS) [44] were employed to solve the redundancy and collinearity problems and improve the accuracy of sub-models. However, sharing a wavelength combination for all sub-models may harm the diversity of the sub-model pool. In our previous studies, it has been found that the weights of the neural networks can be regarded as a self-trained attention mechanism and indicate the importance of wavelengths [53]. In the present work, we utilized the weight indicator for probabilistic wavelength selection, founding the best trade-off between the accuracy and the diversity of the sub-models.

Two infant formula data sets with typical adulterants, namely HLP and melamine, were evaluated to validate the proposed ensemble CNN algorithm based on attention mechanism (named as AM-ECNN). Besides, three classical regression methods (PLS, kernel PLS and CNN) and a frequently-used ensemble learning method (random forest, RF) were also adopted for the purpose of comparison. Moreover, the regression coefficient obtained by the PLS models was also employed for probabilistic wavelength selection and compared with the proposed weight indicator method.

## 2. Materials and Methods 

### 2.1. Samples

Infant formula, as a suitable alternative to breast milk, is a widely consumed and highly concerned powdered dairy product. In the present work, samples for two infant formula adulteration data sets were prepared. The first data set is HLP adulteration [54], consisting of 100 infant formula samples with concentrations of HLP ranged from 0% to 20%. The samples in this data set contained infant formula powder of three brands: Wyeth (Madison, NJ, USA), Mead Johnson (Chicago, IL, USA) and Beingmate (Hangzhou, China), and their mixtures with random proportions. The HLP powder purchased from three producers (Kaitai, Beijing, China; Cargill, Hamburg, Germany; AccoBio, Wuxi, China) was mixed into infant formula to form adulteration samples. Such an experimental design was aimed to improve the robustness of the NIR model to the variations in chemical composition of infant formula and HLP powder in practical use. All of the adulteration samples were well stirred before NIR spectroscopy measurement.

The second data set is melamine adulteration, consisting of 100 infant formula samples with concentrations of melamine ranged from 0% to 10%. Same as the HLP data set, the infant formula powder in the melamine adulteration data set contained infant formula of three brands: Aptamil (Rotterdam, The Netherlands), Wyeth (Vevey, Switzerland) and Frisolac Prestige (Amersfoort, The Netherlands), and their mixtures with random proportions. The melamine powder was purchased from Kermel (Tianjin, China) and well mixed with the infant formula powder before the measurement.

### 2.2. NIR Spectroscopy Measurement 

The NIR spectra were acquired with a handheld NIR analyzer (DLP NIRscan Nano: Texas Instruments, Dallas, TX, USA). The spectra ranged from 900 to 1700 nm (1100–5880 cm^−1^) with a scanning resolution of 2.8 nm. Sixteen diffuse reflectance scans were averaged for each spectrum. All of the measurements were taken at room temperature (24–27 °C) and relative humidity between 50% and 65%.

### 2.3. Wavelength Selection Based on Attention Mechanism 

In the proposed ensemble architecture, an artificial neural network (ANN) was first trained to obtain an attention curve, which is used for the wavelength selection of the sub-models. All of the training samples and all of the NIR variables were fed into the ANN with two hidden layers. The trained weights of the first hidden layer were used to calculate the attention indicator *T*:

(1) $$T_{b}=\sum_{i=1}^{n}\left|w_{i}^{b}\right|,b=1,2,3,\ldots \ldots m,\;$$

where $w_{i}^{b}$ represents the weights corresponding to the *b*th wavelength and the *i* hidden node, *n* is the total number of the hidden node in this layer, *m* is the total number of wavelengths. For the weights of this hidden layer with a shape of *n × m*, *T* calculates the sum of their absolute values for each wavelength. A large value of *T* indicates that this wavelength is highlighted by the ANN and has a greater contribution on the outputs of subsequent layers. 

To ensure sufficient diversity of the sub-models, instead of simply picking important wavelengths with large *T*, the attention indicator *T* was normalized into a probability distribution $T_{nor}$ and used for the probabilistic wavelength sampling of each sub-model.

(2) $$T_{b,\;nor}=\frac{T_{b}}{\sum_{b=1}^{m}T_{b}},\;$$

Such probabilistic wavelength selection strategy ensures that the important bands have greater probabilities of being selected and at the same time keeps the randomness of wavelength combination, as a consequence, achieving a good compromise between the accuracy and the diversity of the sub-models.

### 2.4. Convolutional Neural Network 

With the fast development of deep learning technology in recent years, CNN has been proved to be a powerful tool for representing complicated data and learning features of multiple levels. In this section, we describe the basic theory of CNN and the training details of the CNN sub-models in AM-ECNN.

The CNN architecture adopted in the proposed ensemble learning method is a fully-convolutional network, which is stacked by convolution layers without the usage of fully-connected layer. In the convolution layer, the spectral features of the previous layer are convolved by the learnable convolutional filter, followed by the nonlinear activation. The operation of the *l*th convolution layer is:

(3) $$F^{l}=g\left(F^{l-1}{\;*\;w}^{l}+b^{l}\right)$$

(4) $$g\left(x\right)=\mathrm{LeakyReLU}\left(x\right)=\{\begin{array}{c} x,\;\mathrm{if}\;x>0\\ \;\frac{x}{a},\;\mathrm{if}\;x\;\leq 0 \end{array}$$

where $F^{l-1}$ indexes the feature map in the previous layer and $F^{l}$ presents the feature map of the current layer, $w^{l}$ and $b^{l}$ are the trainable weight and bias of the *l*th convolution layer, * represents the convolution operation. g(x) is the nonlinear activation of the convolution layer which is LeakyReLU in this paper. LeakyReLU is a nonlinear function that simply passes the positive input, while divides the negative inputs by a fixed parameter in range (1, +∞) [55]. Batch normalization layer, which helps to speed up the training process [56], was used before each convolution layer.

The output features are fed into the output layer for prediction. The output layer can be seen as a fully-connected layer with one hidden node. The operation of the output layer can be presented as:

(5) $$P=\sigma \left(\sum_{j=1}^{m}F_{j}\times w_{j}+b\;\right)$$

(6) $$\sigma \left(x\right)=\mathrm{sigmoid}\left(x\right)=\;\frac{1}{1+e^{-x}}$$

where P represents the output prediction, $F_{j}\;\left(j=1,2,3,\ldots ,\;m\right)$ represents the output features obtained by the previous layers, m is the feature map size, $w_{j}$ and b represent the trainable weight and bias of the output layer, σ(x) is the nonlinear activation of the output layer, which is sigmoid for regression problems.

Before the final ensemble-CNN model was built, the number of the convolution layers in the sub-models was optimized in range 1~5, while the number of filters for each convolution layer was optimized in range (8, 16, 32, 64) by an internal cross-validation procedure. During the parameter optimization process, 25 wavelengths were selected by the *T* curve and used to build models. For both data sets, the optimized number of the convolution layers and the number of filters for each convolution layer are 3 and 16, respectively. It was also observed that the usage of pooling layer or convolution layer with stride greater than one deteriorates the accuracy of the sub-models. Since the sub-models have much fewer input wavelengths compared with the full-spectrum model, the pooling operation or large convolutional stride may cause obvious information loss. The detailed structural parameters of the CNN sub-models in AM-ECNN are listed in Table 1. In the training process, the learning rate was 0.0001 for both adulteration data sets, and the training iterations were set to 2000 and 2500 for the HLP and the melamine data sets respectively, in order to gain convergent results. No mini-batch strategy was used in the training process because the size of training samples is small.

### 2.5. Architecture of the AM-ECNN

**Figure 1:**
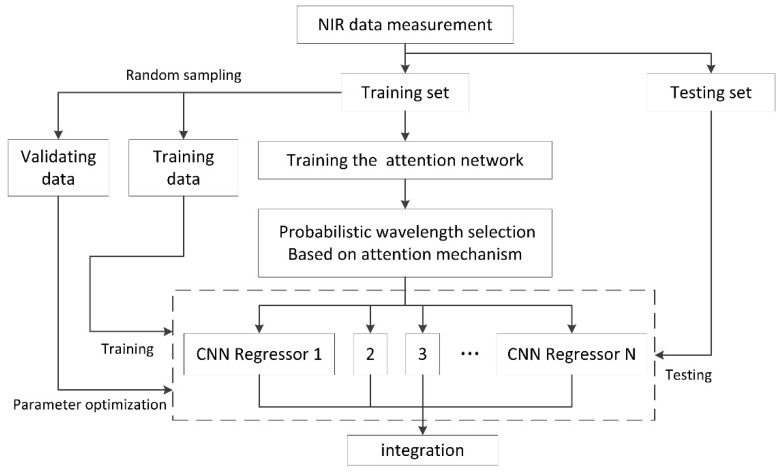
Architecture of the proposed attention mechanism ensemble convolutional neural network (AM-ECNN).

In the present work, an ensemble CNN algorithm based on attention mechanism was developed to improve the performance of infant formula adulteration detection. As shown in Figure 1, the proposed AM-ECNN model was constructed as follows: (1) divided the samples into the training set and the testing set; (2) trained the attention network with all the samples in the training set and all the wavelengths as inputs; (3) normalized the weight indicator obtained by the attention network into a probability distribution and selected the input wavelengths of each sub-model according to it; (4) randomly selected samples in the training set for sub-model construction, while the rest samples were used as the validation data for parameter optimization; (5) trained the CNN sub-models based on the variable and sample selection in steps 3 and 4; (6) fed the testing samples into the trained sub-models and integrated the results for final prediction. 

Two structural parameters for the ensemble architecture, including the number of input wavelengths ($n_{w}$) and the ratio of training samples ($r_{s}$) of each sub-model, were optimized by the prediction results of the validation samples in 10-fold cross-validation. In the integration step, a simple average strategy was used to obtain the final prediction result of the testing samples.

### 2.6. Methods for Comparison 

PLS: PLS is a classical chemometrics algorithm for quantitative analysis of NIR data [57]. In the present work, the number of latent variables used in PLS model was determined in a range of 3~25 by a 5-fold cross-validation within the training sets.

Kernel PLS: Kernel PLS is an improved PLS method that the original input data are projected onto a higher dimensional feature space by kernel functions, in order to deal with the nonlinear problem [58]. The number of latent variables used in the kernel PLS was also optimized in a range of 3~25.

CNN: Regular 1D CNN models were also established for comparison. A typical CNN architecture was adopted, which was stacked by one dimensional convolution layers and pooling layers. A fully convolutional network without fully-connected layers was adopted to reduce the number of parameters and avoid overfitting. The structural parameters of the CNN models, including the number of convolution layers and the number of filters for each convolution layer, were optimized. The detailed information of the optimized CNN for both data sets is listed in Table S1.

RF: Random forest (RF) is a popular ensemble method that consisting of many trees [59]. In the present work, RF models were developed for comparison and two parameters of the RF models, including the number of trees and the maximum depth of the trees, were optimized.

### 2.7. Data Processing, Model Optimization and Evaluation

In order to improve the calibration accuracy, the initial and terminal sections of 100 nm of the spectra were deleted because rather high instrument noise could be observed at these regions. Therefore, the spectra ranging from 1000 nm to 1600 nm were used for analysis. A pretreatment of first-order Savitzky–Golay derivative (smoothing points = 9, polynomial order = 2) was performed on all of the NIR spectra for base line correction and smoothing purpose.

In order to fairly evaluate the performance of the models, an external cross-validation and an internal cross-validation were conducted. The external 10-fold cross-validation was used for final model evaluation, in which the samples were shuffled and divided into ten partitions. In each fold of the external cross-validation, nine sample partitions (90 samples) were used for model training while one sample partition (10 samples) was adopted as an independent testing set. The average and standard deviation of the root mean squared error (RMSEP), the determination coefficient (R^2^) and the ratio of prediction (RPD) of the test sets obtained by the 10-fold external cross-validation were used to evaluate the prediction capacity of each regression model. The RPD can be calculated as:

RPD = SD/RMSEP,(7)

where SD is the standard deviation of the test set in each fold. 

Before the external cross-validation was carried out, the internal cross-validation process was conducted within the training sets of each fold for the purpose of parameter optimization, to ensure that all the methods in comparison were fully optimized. In the 5-fold internal cross-validation process, the samples in training set were divided into five partitions, and five models were built alternately. The parameter optimizations for PLS, kernel PLS, RF were performed before each external model was built, whereas the optimizations (internal cross-validation) for the regular CNN and the sub-models of AM-ECNN were conducted on one of the external fold and the optimized setting was followed by the left external folds, because the cross-validation of the CNN-based method is rather time-consuming. It should also be noticed that for the proposed AM-ECNN, the basic structural parameters of the sub-model were first optimized through the internal cross-validation, and then two key parameters of the ensemble learning architecture (the number of input wavelengths and the ratio of training samples) were optimized through the process described in Section 3.3. 

In this study, all of the CNN-based methods were conducted on the open source platform TensorFlow, the PLS and RF models were performed in scikit-learn, which is a machine learning toolbox in python, and the kernel PLS models were performed in the chemometric software The Unscrambler ver. 10.4 (CAMO, Oslo, Norway).

## 3. Results and Discussion

### 3.1. PCA Analysis 

**Figure 2:**
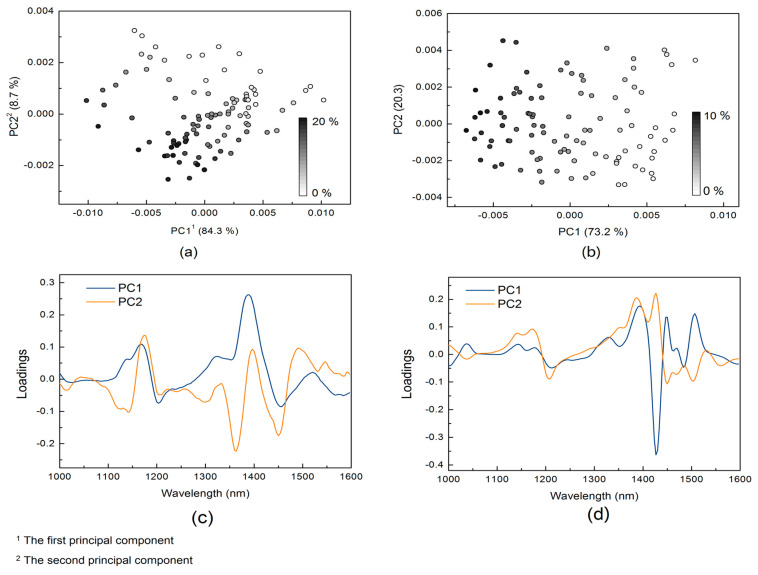
Principal component analysis score plots and loading curves of the infant formula adulteration data sets: (**a**) score plot of the hydrolyzed leather protein (HLP) data set; (**b**) score plot of the melamine data set; (**c**) loading curve of the HLP data set; (**d**) loading curve of the melamine data set.

PCA was performed based on the pre-treated NIR spectra of two adulteration data sets to evaluate the potential of quantitative analysis. The first and second principal component plots for the HLP and the melamine data sets are illustrated in Figure 2a,b respectively, and the contents of adulterants are marked by color gradient. For both studied adulteration data sets, PC1 and PC2 explain about 93.0% of the total variance. For the HLP data set, as shown in Figure 2a, both PC1 and PC2 decrease with the increase of HLP content. On the other hand, for the PCA plot of melamine data set shown in Figure 2b, PC1 clearly indicates the change of melamine content but no obvious change in PC2 can be observed. 

The loading curves of PC1 and PC2 for the HLP and the melamine data sets are illustrated in Figure 2c,d, respectively. The contribution of x-variables for each principal component is proportional to its distance from the origin in the loading space. It can be observed that the melamine data set shows sharper peaks in the loading curves than the HLP data set, indicating the greater discrimination for wavelength importance.

### 3.2. Attention Mechanism Based on the Weight Indicator

**Figure 3:**
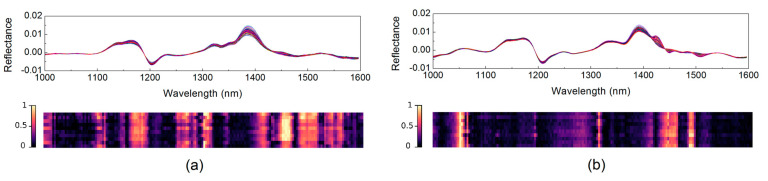
Spectra and weight indicators of the infant formula adulteration data sets: (**a**) hydrolyzed leather protein (HLP) data set; (**b**) melamine data set.

In the proposed ensemble architecture, an ANN was first trained to obtain attention indicators for further wavelength selection of the sub-models. The learned weight indicators *T* obtained by 10-fold cross-validation are demonstrated in the form of heat maps (see in Figure 3), where the horizontal axis represents the wavebands; the vertical axis represents different folds of cross-validation; while the color bar represents the value of weight indicators. The learned *T* curves of the two data sets show highlighted areas, indicating obvious distinction in wavelength importance. For the HLP data set, the highlighted wavelengths concentrate in three regions: 1150~1200 nm, 1250~1320 nm and 1400~1550 nm. When it comes to the melamine data set, the highlighted regions are: 1030~1060 nm, 1306 nm and 1420~1490 nm. 

In general, for both adulteration data sets investigated in the present work, the weight indicator method demonstrated rather good stability that the locations of highlighted wavelengths were basically unchanged for models built by different resampling runs. It can also be observed that the melamine data set showed higher stability and greater discrimination for wavelength importance (sharper and less peaks in the *T* curves) than the HLP data set, which is consistent with the results of PLS loading plots. This may be because melamine is a single substance while HLP is a complex mixture, so it is difficult to simply indicate its content with a few wavelengths. 

**Figure 4:**
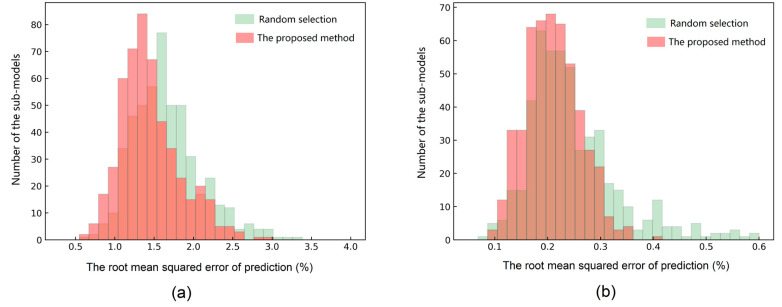
Statistical histograms of the sub-model prediction results obtained by the random selection strategy and the proposed wavelength selection method: (**a**) hydrolyzed leather protein (HLP) data set; (**b**) melamine data set.

The *T* curve was normalized into a probability distribution and used for wavelength selection of each sub-model. To demonstrate the validity of the proposed wavelength selection strategy based on attention mechanism, the prediction accuracy of the sub-models built with the proposed strategy and random selection was compared. The accuracy distribution of 500 sub-models obtained in 10-fold external cross-validation (50 sub-models for each fold) is presented in the form of histogram (see in Figure 4). Each sub-model was trained with all the samples in the training set and 25 NIR features selected by the attention curve or randomly. As shown in Figure 4, for both data sets, the proposed wavelength selection strategy achieved higher accuracies when compared with the random feature selection in general, especially showing the advantage of avoiding sub-models with poor performance. With such a feature selection method, the effective wavelengths have high probabilities of being selected, and at the same time, the diversity of feature combinations is guaranteed.

### 3.3. Parameter Optimization

In this section, we optimized two structural parameters of the ensemble learning architecture: the number of input wavelengths ($n_{w}$) and the ratio of training samples ($r_{s}$) of the sub-models, before the final models were built for the prediction of the testing sets. Considering an effective ensemble method requires sub-models with good accuracy and diversity, we built sub-models and adopted the root mean square error (RMSEV) and the mean standard deviation (MSDV) of the validating samples for the parameter evaluation. The MSDV is defined as:

(8) $$\mathrm{MSDV}=\frac{1}{m}\frac{1}{\sqrt{n}}\sum_{i=1}^{m}\sqrt{\sum_{j=1}^{n}{\left({\hat{y}}_{i}^{j}-{\bar{y}}_{i}\right)}^{2}}$$

where ${\hat{y}}_{i}^{j}$ represents the prediction result of the *i*th validation sample obtained by the *j*th sub-model, ${\bar{y}}_{i}$ represents the average prediction value of the *i*th validation sample, *n* is the total number of the sub-models in which this sample is used for validation, *m* is the total number of validation samples. In all the experiments, the number of sub-models was fixed to 50, which is relatively small because the training of CNN is more time-consuming than the traditional methods.

**Figure 5:**
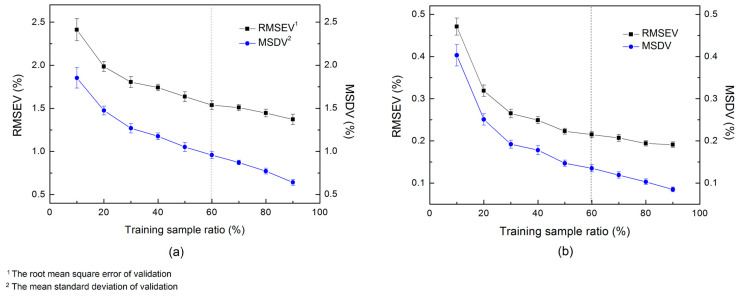
Optimization of the training sample ratio ($r_{s}$) for the infant formula adulteration data sets: (**a**) hydrolyzed leather protein (HLP) data set; (**b**) melamine data set.

Firstly, the $n_{w}$ was set to 25, and the $r_{s}$ varied in the range of 10~90%. Each Sub-model was built with 25 input wavelengths selected by the attention probability distribution and evaluated by the validating samples. The RMSEV and MSDV obtained by 10-fold external cross-validation were illustrated in Figure 5. It is not surprised that both the RMSEV and the MSDV decrease with the increase of training sample size. It can be seen that the RMSEV and the MSDV decline sharply first, and the downward trend has leveled off as the sample size continues to increase, especially in the melamine data set. To balance the accuracy and the diversity, we set the hyper-parameters $r_{s}$ to 60% for both studied data sets, where the variation of accuracy is relatively flat and the diversity is maintained at a certain degree.

**Figure 6:**
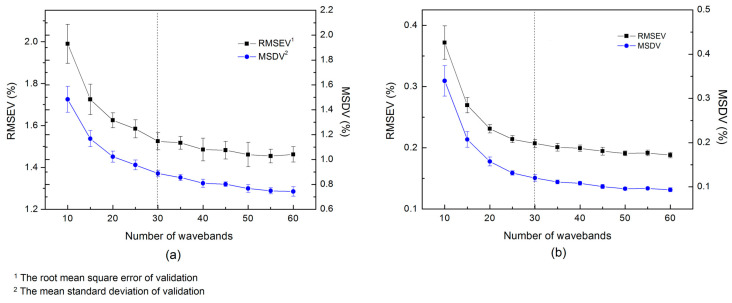
Optimization of the number of wavelengths ($n_{w}$) for the infant formula adulteration data sets: (**a**) hydrolyzed leather protein (HLP) data set; (**b**) melamine data set.

Then, sub-models were built with the $n_{w}$ varied in the range of 10~60 and the $r_{s}$ fixed to 60%. As can be seen in Figure 6, similar to the variation of sample size, the RMSEV and MSDV obtained by 10-fold cross-validation decline sharply with the increase of input wavelengths first, and then the plateaus appear as $n_{w}$ continues to increase. Therefore, we set the hyper-parameters $n_{w}$ to 30 for both data sets since the accuracy and diversity start to stabilize under this parameter setting. It should also be noticed that under the same CNN structure (depth and width of the network), more input features also increase the number of CNN weights, which brings a higher risk of overfitting and greater training time consumption to each sub-model. As a consequence, a relatively small $n_{w}$ was chosen for the proposed ensemble learning architecture. 

### 3.4. Results Comparison

**Figure 7:**
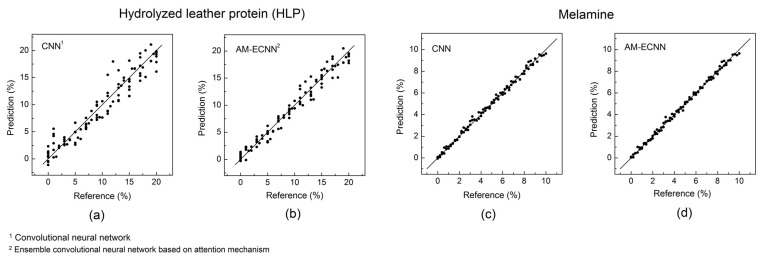
Regression plots obtained by the convolutional neural network (CNN) and the ensemble convolutional neural network based on attention mechanism (AM-ECNN) (**a**) CNN regression plot for the hydrolyzed leather protein (HLP) data set; (**b**) AM-ECNN regression plot for the HLP data set; (**c**) CNN regression plot for the melamine data set; (**d**)AM-ECNN regression plot for the melamine data set.

The proposed AM-ECNN models were established for the prediction of the testing data, after the parameter optimization process. For a comparison, the regression coefficient obtained by the PLS models was also adopted for probabilistic wavelength selection to construct ensemble CNNs. In the PLS models, wavelengths with large absolute values of regression coefficient represent more importance on predicting y-variable. Therefore, the absolute values of regression coefficient were also normalized to a probability distribution for wavelength selection, and the corresponding ensemble CNN network was denoted as RC-ECNN. In Table 2 and Table 3, the root mean squared error of prediction (RMSEP), the determination coefficient of the testing samples (R^2^) and the ratio of prediction (RPD) of the HLP and the melamine data sets were summarized. It can be clearly observed in these two tables that the ensemble CNN models outperform the PLS, kernel PLS, CNN and RF models, and the AM-ECNN models always provide the best performances of RMSEP, R^2^ and RPD for both the HLP and the melamine adulteration data sets. The average accuracy gains of two data sets obtained by the AM-ECNN are 33.3%, 27.8%, 22.5%, 44.4% and 3.9% respectively, when compared with the PLS, kernel PLS, CNN, RF and RC-ECNN models. The regression plots of the regular CNN and the AM-CNN are illustrated in Figure 7, while the regression plots of other contrast methods can be found in Figure S1.

As for the comparison of the two infant formula adulteration data sets, the melamine adulteration data set (best R^2^ = 0.995) possesses much higher detection accuracy than the HLP data set (best R^2^ = 0.961). As mentioned above, HLP is a complex mixture and its chemical composition varies with different extraction techniques and raw materials (leather scraps). Besides, compared with the melamine, the chemical composition and the NIR characteristics of HLP are more similar to those of milk powder. Therefore, as a new type of adulterant, HLP needs more research to obtain its accurate and robust determination by NIR spectroscopy. 

## 4. Conclusions

An ensemble CNN method was developed based on attention mechanism for NIR data processing, called AM-ECNN, and it was evaluated on two infant formula adulteration data sets. In the proposed algorithm, the learned weight of ANN was regarded as a self-trained attention mask and used as the probability distribution for wavelength selection, then the convolutional sub-models were built and the results were integrated for the final prediction. Compared with the random wavelength selection, the proposed wavelength selection method achieves a rather good balance between accuracy and diversity of the sub-models, especially avoiding poorly performing sub-models. The prediction results demonstrated that the proposed ensemble method yielded superior regression performance compared with the PLS, kernel PLS, CNN, RF and RC-ECNN, for both the HLP and the melamine adulteration data sets. 

However, as is known, a robust NIR model for adulteration detection in milk powder is hard to achieve due to multiple variation factors, such as different brands and batches of milk powder, simultaneous existence of several adulterants, temperature, humidity and spectral drift of light sources, making it hard to obtain stable applications in practice. Therefore, more investigations should be carried out to evaluate and improve the robustness of the proposed AM-ECNN method. The limitations of the proposed method should also be further considered and improved. For example, compared with the traditional method and the regular convolutional network, the ensemble method is much more time-consuming in training. Thanks to the fast development of deep learning hardware, graphics processing unit (GPU) for instance, the testing time for the proposed network is acceptable. Another concern is that although this method proposes a wavelength selection method, it cannot be applied to the multispectral system, because each wavelength has a probability to be selected by the sub-models during the wavelength selection process. In conclusion, the overall results demonstrate that the AM-ECNN is a promising quantitative analysis method of determining adulteration in infant formula by using NIR spectroscopy.

## Figures and Tables

**Table 1:** The detailed information of sub-models in ensemble convolutional neural network based on attention mechanism (AM-ECNN) for both data sets.

Layers	Number of Filters	Kernel Size	Stride	Padding	Nonlinear Activation
Convolution Layer 1	16	5	1	Yes	LeakyReLU
Convolution Layer 2	16	5	1	Yes	LeakyReLU
Convolution Layer 3	16	5	1	Yes	LeakyReLU
Output	/	/	/	/	Sigmoid

**Table 2:** Regression results of the hydrolyzed leather protein (HLP) data set obtained by the ensemble convolutional neural network based on attention mechanism (AM-ECNN) and the contrast methods.

	RMSEP	R^2^	RPD
PLS ^1^	1.600 ± 0.295	0.930 ± 0.025	4.157 ± 0.728
Kernel PLS ^2^	1.444 ± 0.254	0.949 ± 0.021	4.933 ± 1.085
CNN ^3^	1.546 ± 0.366	0.933 ± 0.029	4.412 ± 1.197
RF ^4^	1.780 ± 0.393	0.911 ± 0.042	3.789 ± 0.813
RC-ECNN ^5^	1.225 ± 0.238	0.957 ± 0.019	5.548 ± 1.516
AE-ECNN ^6^	1.168 ± 0.231	0.961 ± 0.016	5.804 ± 1.818

^1^ Partial least squares regression; ^2^ Kernel partial least squares regression; ^3^ Convolutional neural network; ^4^ Random forest; ^5^ Ensemble convolutional neural network based on regression coefficient; ^6^ Ensemble convolutional neural network based on attention mechanism.

**Table 3:** Regression results of the melamine data set obtained by the AM-ECNN and the contrast methods.

	RMSEP	R^2^	RPD
PLS ^1^	0.263 ± 0.058	0.987 ± 0.010	11.138 ± 4.052
Kernel PLS ^2^	0.250 ± 0.044	0.992 ± 0.008	12.377 ± 4.826
CNN ^3^	0.200 ± 0.041	0.992 ± 0.005	14.973 ± 6.975
RF ^4^	0.349 ± 0.093	0.980 ± 0.011	8.244 ± 2.235
RC-ECNN ^5^	0.164 ± 0.032	0.995 ± 0.004	17.811 ± 6.228
AE-ECNN ^6^	0.159 ± 0.028	0.995 ± 0.004	18.004 ± 5.662

^1^ Partial least squares regression; ^2^ Kernel partial least squares regression; ^3^ Convolutional neural network; ^4^ Random forest; ^5^ Ensemble convolutional neural network based on regression coefficient; ^6^ Ensemble convolutional neural network based on attention mechanism.

## Data Availability

Data sharing not applicable.

## Supplementary Materials

The following are available online at https://www.mdpi.com/article/10.3390/foods10040785/s1, Figure S1: Regression plots obtained by the contrast methods (a) partial least squares regression (PLS) regression plot for the hydrolyzed leather protein (HLP) data set; (b) kernel partial least squares regression (Kernel PLS) regression plot for the HLP data set; (c) random forest (RF) regression plot for the HLP data set; (d) ensemble convolutional neural network based on regression coefficient (RC-ECNN) regression plot for the HLP data set; (e) PLS regression plot for the melamine data set; (f) Kernel PLS regression plot for the melamine data set; (g) RF regression plot for the melamine data set; (h) RC-ECNN regression plot for the melamine data set, Table S1: The detailed information of the regular convolutional neural network (CNN) for both data sets.
