# Supplementary material for: Detection of Adulteration in Infant Formula Based on Ensemble Convolutional Neural Network and Near-Infrared Spectroscopy
Source: Foods. 2021 Apr 6;10(4):785. doi: 10.3390/foods10040785 (PMC8067368; doi:10.3390/foods10040785)
Supplement: Supplementary file 1 [file foods-10-00785-s001.pdf]

Response to comments

Dear Editors and Reviewers:

Thank you for your letter and for the Reviewer's comments concerning our manuscript entitled "**Detection of adulteration in infant formula based on ensemble convolutional neural network and near-infrared spectroscopy (manuscript ID: foods-1155110)**". Those comments are all valuable and very helpful for revising and improving our paper, as well as the important guiding significance to our researches. We have made revisions to take into consideration the Reviewer's comments. The main corrections in the paper and the response to the Reviewer's comments are as following:

Reviewer 3:

I can see that the authors have made huge efforts to improve the manuscript, yet I am afraid it is still not adequate.

(1) Table 1 shows that for the stacked three convolution layers, the parameters (kernel size, stride) are exact the same, which is not common. I would like to see the input size for each convolution layer and the output size (i.e. activation) from each layer, as an example shown as below:

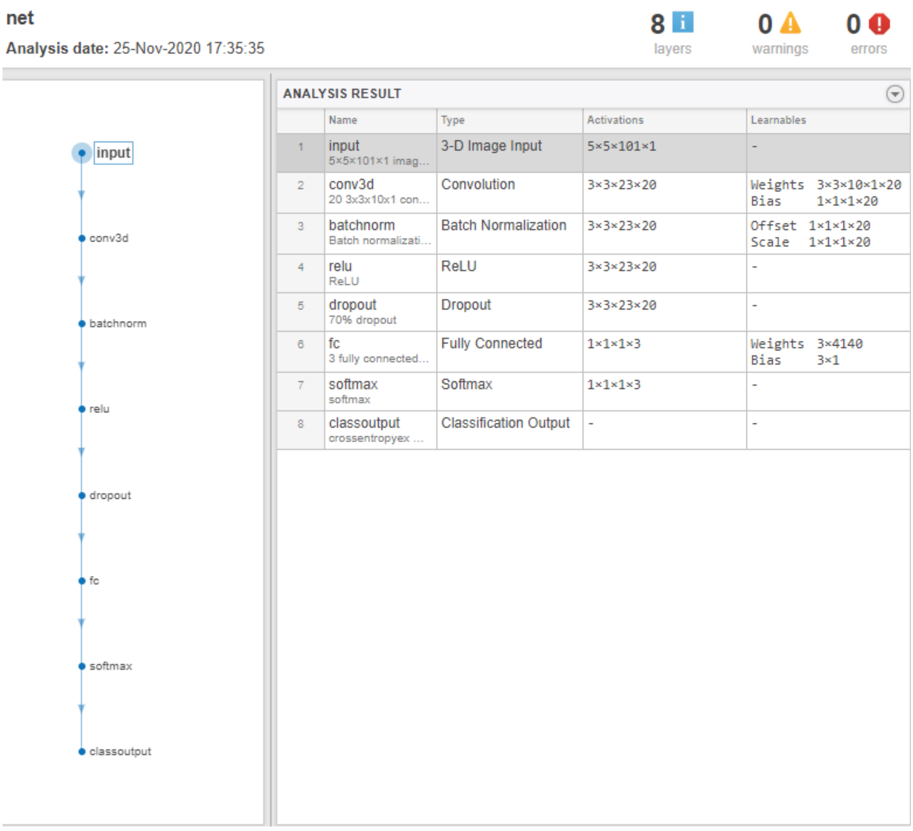

	Name	Type	Activations	Learnables
1	input<br>5×5×101×1 imag...	3-D Image Input	5×5×101×1	-
2	conv3d<br>20 3x3x10x1 con...	Convolution	3×3×23×20	Weights 3×3×10×1×20<br>Bias 1×1×1×20
3	batchnorm<br>Batch normalizati...	Batch Normalization	3×3×23×20	Offset 1×1×1×20<br>Scale 1×1×1×20
4	relu<br>ReLU	ReLU	3×3×23×20	-
5	dropout<br>70% dropout	Dropout	3×3×23×20	-
6	fc<br>3 fully connected...	Fully Connected	1×1×1×3	Weights 3×4140<br>Bias 3×1
7	softmax<br>softmax	Softmax	1×1×1×3	-
8	classoutput<br>crossentropyex ...	Classification Output	-	-

In addition, in Table 1, the filter size is equal to kernel size, I guess the authors meant the number of filers here. Correction is needed.

Response: According to the suggestion of the Reviewer, we have listed the sizes of weights, inputs and outputs of each layer in the Table below.

Table. The weight and feature sizes of the CNN sub-models.

Layers	Number of Filters	Kernel Size	Stride	Padding	Nonlinear Activation	Weight Size	Input Feature Size	Output Feature Size
Conv Layer 1	16	5	1	Yes	LeakyReLU	Weights: 5×1×16<br>Bias: 1×16	30×1	30×16
Conv Layer 2	16	5	1	Yes	LeakyReLU	Weights: 5×16×16<br>Bias: 1×16	30×16	30×16
Conv Layer 3	16	5	1	Yes	LeakyReLU	Weights: 5×16×16<br>Bias: 1×16	30×16	30×16
Output Layer	/	/	/	/	Sigmoid	Weight: 480×1<br>Bias: 1×1	480	1

In term of the selection of the kernel size and the convolutional stride of the CNN sub-models, we had made some observation on the parameter optimization stage. For the convolutional stride, we found that the usage of pooling layer or convolution layer with stride greater than one would bring damage to the accuracy of the sub-models. This maybe because the sub-models have much fewer input wavelengths when compared with the full-spectrum models, therefore, the pooling operation or large convolutional stride may cause information loss to the sub-models. Therefore, we set stride=1 and did not use any pooling layer for all the sub-models. We have summarized the sub-model accuracies obtained by the validation samples in 10-fold cross validation to show the effect of the convolutional stride.

Table. The RMSECVs obtained by the sub-models with different convolutional strides.

Strides of the three Conv layers	2/2/2	2/2/1	2/1/1	1/1/1 (the present work)
RMSECV (HLP data set)	1.750±0.054	1.746±0.053	1.707±0.046	1.525±0.041
RMSECV (Melamine data set)	0.237±0.024	0.231±0.007	0.224±0.006	0.207±0.007

When it comes to the selection of the kernel size, we have observed in the optimization stage that it was not a crucial parameter for the performance of the sub-models. Therefore, we simply set the kernel size to 5 based on our experience on spectral modelling. Here, we also summarized the sub-model accuracies obtained by the validation samples in 10-fold cross validation with different kernel sizes.

Table. The RMSECVs obtained by the sub-models with different kernel sizes.

Kernel sizes of the three Conv layers	3/3/3	5/5/5 (the present work)	7/7/7	3/5/7
RMSECV (HLP data set)	1.618±0.053	1.525±0.041	1.571±0.037	1.590±0.046
RMSECV (Melamine data set)	0.202±0.004	0.207±0.007	0.204±0.005	0.205±0.008

To make the parameter optimization part more clear for the readers, we have added the following sentences in the revised manuscript, Page 5, Line 205-208:

"It was also observed that the usage of pooling layer or convolution layer with stride greater than one deteriorates the accuracy of the sub-models. Since the sub-models have fewer input wavelengths compared with the full-spectrum model, the pooling operation or large convolutional stride may cause obvious information loss."

In Table 1, 'filter size' did mean 'number of filters'. We apologized for the incorrect use of term and have made corrections in **Table 1**, **Table S1**, and **the revised manuscript, Page 5, Line 201 and 204**.

Table 1. The detailed information of the CNN sub-models in AM-ECNN for both data sets.

Layers	Number of Filters	Kernel Size	Stride	Padding	Nonlinear Activation
Convolution Layer 1	16	5	1	Yes	LeakyReLU
Convolution Layer 2	16	5	1	Yes	LeakyReLU
Convolution Layer 3	16	5	1	Yes	LeakyReLU
Output	/	/	/	/	Sigmoid

Besides, we have carried out a thorough English examination and made some corrections. For instance:

(1) In the revised manuscript, Page 4, Line 184, changed "…while divide the negative inputs…" to "…while divides the negative inputs…".

(2) In the revised manuscript, Page 11, Line 380, changed "…wavelengths with large absolute values of regression coefficient represents more…" to "…wavelengths with large absolute values of regression coefficient represent more…".

(3) In the revised manuscript, Page 12, Line 419, changed "…yielded superiority regression performance…" to "…yielded superior regression performance…".

We have tried our best to improve the manuscript and made corrections in the manuscript according to the suggestion of the reviewers. These changes have been marked in red in revised paper. We appreciate for Editors/Reviewers' warm work earnestly, and hope that the correction will meet with approval. Once again, thank you very much for your comments and suggestions.

Change list

(1) In the revised manuscript, Page 4, Line 184, changed "…while divide the negative inputs…" to "…while divides the negative inputs…".

(2) In the revised manuscript, Page 5, Line 201 and 204, changed "…the filter size of each convolution layer…" to "…the number of filters for each convolution layer …".

(3) Added in the revised manuscript Page 5, Line 205-208: "It was also observed that the usage of pooling layer or convolution layer with stride greater than one deteriorates the accuracy of the sub-models. Since the sub-models have much fewer input wavelengths compared with the full-spectrum model, the pooling operation or large convolutional stride may cause obvious information loss."

(4) In the revised manuscript, Page 6, Line 245, changed "…the filter size of each convolution layer…" to "…the number of filters for each convolution layer …".

(5) In the revised manuscript, Page 11, Line 380, changed "…wavelengths with large absolute values of regression coefficient represents more…" to "…wavelengths with large absolute values of regression coefficient represent more…".

(6) In the revised manuscript, Page 12, Line 419, changed "…yielded superiority regression performance…" to "…yielded superior regression performance…".

(7) Changed "Filter size" to "Number of Filters" in the revised Table 1:

Table 1. The detailed information of the CNN sub-models in AM-ECNN for both data sets.

Layers	Number of Filters	Kernel Size	Stride	Padding	Nonlinear Activation
Convolution Layer 1	16	5	1	Yes	LeakyReLU
Convolution Layer 2	16	5	1	Yes	LeakyReLU
Convolution Layer 3	16	5	1	Yes	LeakyReLU
Output	/	/	/	/	Sigmoid

(8) Changed "Filter size" to "Number of Filters" in the revised Table S1:

Table S1. The detailed information of the regular CNN for both data sets.

CNN for the HLP data set					
Layers	Number of Filters	Kernel Size	Stride	Padding	Nonlinear Activation
Convolution Layer 1	16	5	2	Yes	LeakyReLU
Convolution Layer 2	16	5	2	Yes	LeakyReLU
Convolution Layer 3	16	5	2	Yes	LeakyReLU
Output	/	/	/	/	Sigmoid
CNN for the melamine data set					
Layers	Number of Filters	Kernel Size	Stride	Padding	Nonlinear Activation

Convolution Layer 1	64	5	2	Yes	LeakyReLU
Convolution Layer 2	64	5	2	Yes	LeakyReLU
Convolution Layer 3	64	5	2	Yes	LeakyReLU
Output	/	/	/	/	Sigmoid
